# Comparative levels and time trends in blood pressure, total cholesterol, Body Mass Index and smoking among Caucasian and South-Asian participants of a UK primary-care based cardiovascular risk factor screening programme

**DOI:** 10.1186/1471-2458-5-125

**Published:** 2005-11-28

**Authors:** Georgios Lyratzopoulos, Patrick McElduff, Richard F Heller, Margaret Hanily, Philip S Lewis

**Affiliations:** 1 Directorate of Public Health and Clinical Services, Norfolk, Suffolk and Cambridgeshire Strategic Health Authority, Cambridge, UK; 2 Hunter New England Population Health, Hunter New England Area Health Service, Wallsend, Australia; 3 Evidence for Population Health Unit, University of Manchester, Manchester, UK; 4 Former Stockport Community Health Trust, Stockport, UK; 5 Department of Cardiology, Stockport NHS Trust, Stockport, UK

## Abstract

**Background:**

Individuals of South-Asian origin have a comparatively higher cardiovascular disease burden, but there is uncertainty about whether this is due to differences in risk factor levels and trends. We therefore studied comparative levels and time trends in blood pressure (BP), total cholesterol, body mass index (BMI) and current smoking among UK Caucasian and South-Asian individuals.

**Methods:**

Repeatable cross-sectional survey of men and women aged 35–60 attending for first screening as part of a primary-care based cardiovascular risk factor screening programme 1989 and 1999.

**Results:**

Of 34,122 men and 37,294 women participants, 499 men (1.5%)and 381 women (1%) were of South-Asian origin. South-Asian men had lower systolic [(-4.91 mmHg (95% Confidence Iterval (CI): -3.58 to -6.23)] and diastolic BP [-2.87 mmHg (-2.02 to -3.72)], with no significant differences in cholesterol and BMI. South-Asian women had lower systolic BP [-1.77 mmHg, 95% (-0.21 to -3.33)], diastolic BP [-1.87 mmHg (-0.92 to -2.82)], cholesterol [-0.24 mmol/l (-0.08 to -0.39)]; and higher BMI [+0.78 kg/m^2 ^(0.25 to 1.3)]. South-Asian men and women had significantly lower prevalence of self-reported current smoking (29.0% and 1.8% respectively). With the exception of self-reported current smoking, between ethnic group risk factor trends were not converging.

**Conclusion:**

With the exception of women's BMI, South-Asian individuals had either lower or similar levels of the examined cardiovascular risk factors, compared with Caucasian individuals. Although time trends in smoking were converging, other risk factors trends were similar between the two ethnic groups. Overall the findings do not support the hypothesis that the relatively high cardiovascular disease burden in UK South-Asians is due to higher levels exposure to the examined risk factors. Other hypotheses, such as higher frequency of diabetes and increased genetic predisposition, require further exploration.

## Background

Cardiovascular disease is the leading cause of death in the UK and responsible for approximately 238,000 deaths annually, or 39% of total mortality [1]. Some UK ethnic groups have a higher cardiovascular disease burden, and this is particularly true for individuals of South-Asian origin [2]. The causes of higher disease burden among individuals of South-Asians origin are not fully understood, but may include excess exposure to known risk factors, excess exposure to as yet unknown risk factors, greater biological susceptibility to cardiovascular disease, or a lower risk of competing mortality and morbidity from other diseases (such as cancer) [2].

In the UK and over the past two decades, the mean population values of total cholesterol and blood pressure have been decreasing, and the same has been true for the prevalence of current smoking; however, mean values of body mass index (BMI) manifest an increasing trend [3]. Whilst cardiovascular risk factor levels for the UK population, including for ethnic groups [4], are relatively well described, there is a relative paucity of information about cardiovascular risk factor time trends in UK ethnic groups, particularly for the period before 1999. Evidence about recent trends in cardiovascular risk factors in UK individuals of South-Asian origin is therefore required [5].

Although a previous meta-analysis of primary studies reported between 1977 and 1996 has questioned the overall efficacy of multiple cardiovascular risk factor screening as a means of preventing the development of cardiovascular disease[6], one potentially important secondary use of population-based screening programmes is as public health surveillance tools, to monitor population risk factor trends. The importance of best using information from routine sources to help give a comprehensive picture of population health and its determinants has been highlighted recently by both the most recent "Wanless Report" [7], and the subsequent UK Department of Health White Paper on Public Health [8]. The Stockport Cardiovascular Disease Risk Factor Screening Programme, originally introduced in 1989, provides an example for the potential secondary use of routine data sources for public health surveillance purposes [9]. Further details about operating protocols and population coverage have been previously described [10,11]. Briefly the Programme was introduced in 1989, and used a call-recall system operated by the Stockport Health Authority, where all Stockport residents aged between 35 and 60 were invited every five years to book a screening appointment at their GP surgery. Cardiovascular risk factors including blood pressure, total cholesterol, BMI and smoking status were assessed, usually by a practice nurse. Between 1989 and 1993, about 10.8% of all patients registered with a GP were excluded from the screening invitation, as they were already known to suffer from hypertension (3.9%), diabetes (1.2%) and conditions including history of any cardiovascular disease, and terminal illness (6.6%).10 Over the whole 11-year period 1989–1999, the population coverage for one (first) screening was about 72.2% for Stockport men aged 35–60 and about 78.4% for Stockport women (see Additional File 1). Individual data on risk factor levels were collated by the Health Authority and anonymised into a usable electronic dataset, which was used in the present study [10,11]. We therefore conducted a study to examine recent risk factor levels and risk factor trends in cardiovascular risk factors among individuals of Caucasian and South Asian origin in a UK population using the Stockport Cardiovascular Risk Factor Screening Programme dataset 1989–1999.

Stockport is a borough of Northwest England. In recent years, general population health has been slightly better than the general UK population, with a Standardised Mortality Ratio from all causes (all ages) of 96 (95% CI 94–98)[12]. In the 1991 Census 1.12% of all Stockport residents were of South Asian origin, and this proportion rose to 2.1% in the 2001 Census [13].

## Methods

Information on 34,122 men and 37,294 women who had a first screening episode during the 11-year period 1989–1999 was analysed.

### Attribution of ethnicity

Information on ethnicity was imputed by the use of the Nam Pahcham software programme version 1.1 [14]. For data protection purposes, the process of assigning ethnic group was completely separated by the data held on each individual, although anonymous linkage occurred at a later stage, by the use of a key identifier, by Stockport Primary Care Trust, who were the legal custodians of the data. The programme was run against the full list of surnames of people included in the database, in order to identify individuals belonging to non-Caucasian ethnic groups. Adopting a method originally described by Cummins et al. [15] the initial yield of names identified as belonging to ethnic group individuals was subsequently visually inspected for inaccuracies by two investigators independently. One of the investigators was of Caucasian and one of South-Asian descent, and results were compared before a final decision was made as to whether names identified as belonging to individuals of ethnic background corresponded to South-Asian individuals. Subsequently the same pair of investigators independently visually inspected all names not initially identified as of probably ethnic origin, to identify any "false negatives". A person was assigned to the South-Asian ethnic group if their name matched the typology of known names of Pakistani, Bangladeshi or Indian origin. Therefore, in this paper South-Asian ethnicity includes a heterogeneous group of persons born either in or outside the UK, and belonging to one of the many ethnic and religious groups originating from Bangladesh, India, Pakistan and Sri Lanka.

### Risk factor measurements

Details are included in Additional File 2, as described previously^.10 ^Briefly, total serum cholesterol was measured at the biochemistry laboratory of the Stepping Hill Hospital (a typical UK District General Hospital) by an enzymatic colorimetric assay using cholesterol esterase and cholesterol oxidase [16]. The Biochemistry laboratory participated in external quality assurance schemes. Fresh blood samples were sent to the laboratory and processed within 24 hours of sampling. Systolic and diastolic BP were measured according to a standardised protocol conforming to the 1987 "Recommendations on Blood Pressure Measurement" of the British Hypertension Society [17]. Training for all physical measurements was provided by a visiting nurse facilitator, employed by the Stockport Health Authority, whose role was to quality assure and co-ordinate the implementation of the screening activity. Smoking status was ascertained by direct questioning (self-reported).

### Statistical analysis

For each ethnic group, simple counts and proportions were calculated for ascertainment completeness of each risk factor.

#### Comparison of risk factor levels

To assess whether there were differences in the average level of a risk factor between ethnic groups, by sex, firstly age-standardised risk factor levels were calculated by the direct method [18-20]. Due to the relatively small number of individuals in the South-Asian group, means for four two-year sub-periods (1989–90, 1991–2, 1993–4, 1995–6) and one three-year sub-period (1997–9) were calculated. Associated 95% confidence intervals were calculated using the normal approximation to the binomial distribution for each age stratum and combining all the strata using weighted averages.

To inform the interpretation of standardised rates, and to examine the significance of differences in risk factor levels between ethnic groups, regression models were constructed. For each continuous risk factor (as the dependent variable), a linear regression model was fitted, with age, test year and ethnic group as the independent variables (Model 1a). In these models, the co-efficient for *ethnic group *denotes the (age- and test year – adjusted) difference in risk factor levels between the Caucasian and South-Asian groups. For smoking, similarly, a logistic regression model was constructed, with current smoking status (current smoker vs. non-smoker) as the dependent binary variable, and age, test year and ethnic group as independent variables (Model 1b). In this model, the exponential of the co-efficient for *ethnic group *denotes the (age- and test year – adjusted) odds ratio of current smoking status in the South-Asian group compared to the Caucasian.

#### Comparison of risk factor trends

To examine changes in risk factor levels over time by ethnic group, for each continuous risk factor (as the dependent variable), a linear regression model was fitted, with age and test year as the independent variables, and the model was fitted for the two ethnic groups separately (Model 2a). Similarly, for smoking, a logistic regression model was constructed, with the binary outcome of current smoking as the dependent variable, and adjusted for age and test year, and applied separately for each ethnic group (Model 2b). The coefficients for *test year *from these models denote the mean annual age-adjusted change in the risk factor level (continuous variables), or mean annual change in the logit of the probability of smoking status, specific to each ethnic group.

Whether trends in risk factors for the two ethnic groups studied were different (e.g. converging) was first examined empirically, by observing whether there was an overlap in the 95% confidence intervals of the *test year *co-efficients for each ethnic group. Secondly, the significance of any differentials in slope over time was assessed with the significance level of the co-efficient for an interaction term *ethnic group *X *test year *which was included in the models already containing the age, test year, and ethnic group variables both for continuous (Model 3a) and the smoking variable (Model 3b). Interaction variables were centred to avoid possible co-linearity.

## Results

There were 34,122 men and 37,294 women aged 35–60 with a first screening episode during 1989 and 1999. Following the steps described below 33,597 men and 36,890 women were assigned Caucasian and 499 men and 381 women were assigned South-Asian origin.

### Attribution of ethnicity

Of all participants, 1.8% were initially identified as of probable South-Asian descent by the Nam Pahcham software. Of all persons initially identified as of probable ethnic origin, 69.2% (or 1.23% of all cases) were judged to truly correspond to South-Asian names. Furthermore 27.3% of individuals initially identified as of ethnic origin (or 0.48% of all cases) were judged as definitely non-South-Asian (Caucasian), 2.1% (or 0.03% of all cases) were judged as Chinese, and 1.4% (or 0.02% of all cases) were judged as of ethnic origin but not certain if South-Asian or other. Subsequent inspection of all surnames not initially identified as of probable ethnic origin by the Nam Pahcham software, identified fewer than 20 (or <0.02% of all cases) additional individuals with a South-Asian (Hindu) surname/origin that were missed out by the software; their ethnicity was re-assigned. Further analysis was restricted to individuals in the Caucasian and South-Asian groups.

### Descriptive comparisons

The mean age of Caucasian individuals (both men and women respectively) was greater than of South-Asian individuals (Table 1). Diastolic blood pressure ascertainment was complete (100%), and systolic blood pressure nearly complete for all groups and both sexes. In women, 68.9% of all first screening episodes occurred during the "prevalence round" of the programme (1989–1993), whilst in men the same proportion was 65.1%. The relative percentages were lower in South-Asian individuals. Ascertainment completeness for BMI, smoking status and cholesterol was systematically lower in South-Asian individuals, as also previously reported [11], although the both the absolute and relative magnitude of this difference was small. There were only small differences over time between ethnic groups in ascertainment completeness for any of the above three variables (Additional File 3, Figures 1-2).

**Table 1:** Main characteristics of participants, by ethnic group

	**Men**		**Women**	
**Baseline characteristic**	**Caucasian (n = 36,890)**	**South-Asian (n = 381)**	**Caucasian (n = 33,597)**	**South-Asian (n = 499)**

**Mean Age (years)**	45.6 (IQR: 39.0–52.0)	44.2 (IQR: 37.0–50.0)	45.7 (IQR: 39.0–53.0)	43.7 (IQR: 38.0–50.0)
**% of first screening episodes curing "prevalence round" 1989–1993**	65.3%	53.3%	69.0%	54.6%
**Ascertainment completeness (%)**				
**BMI**	83.2%	79.4%	80.5%	77.7%
**Total Cholesterol**	58.8%	52.3%	53.5%	47.2%
**Smoking status**	83.7%	79.2%	81.0%	76.1%

IQR: Inter-quartile range, BMI: Body Mass Index.

### Comparisons of risk factor levels

The 95% confidence intervals of values relating to South-Asian individuals however, due to small numbers, often overlap with the confidence intervals of values for Caucasian individuals relating to the same time period. Table 2 below presents summary information showing the difference in risk factor levels between the two ethnic groups, for the two most distant time periods in the study (1989–1990 and 1997–9), along with the proportion ascertained and the number of individuals contributing data to each time period and for each individual risk factor. The most substantial changes in both absolute and relative terms relate to a decline in total cholesterol (for both sexes and ethnic groups) and current smoking status, the latter only for the Caucasian group.

**Table 2:** Summary information about risk factor levels between study baseline (1989–90) and end period (1997–9), by ethnic group. Percentage ascertainment and sample size presented, along with absolute and % difference.

			**Men**				**Women**			
			
			**1989–90**	**1997–9**	**Absolute difference**	**% difference**	**1989–90**	**1997–9**	**Absolute difference**	**% difference**
**Cholesterol**	**Caucasian**	% ascertained	51.7%	50.5%			47.6%	43.8%		
		n	4,096	2,591			4,454	2,130		
		Mean (mmol/l) (95% CI)	6.168 (6.133 – 6.203)	5.628 (5.578 – 5.678)	-0.54	-8.8%	6.034 (6.002–6.066)	5.409 (5.358 – 5.460)	-0.625	-10.4%
	**South-Asian**	% ascertained	40.9%	50.0%			41.8%	45.5%		
		n	38	55			28	35		
		Mean (mmol/l) (95% CI)	6.539 (6.209 – 6.869)	5.614 (5.293 – 5.935)	-0.925	-14.1%	5.629 (5.181–6.077)	5.217 (4.935 – 5.499)	-0.412	-7.3%
**SBP**	**Caucasian**	% ascertained	100%	100%			99.9%	99.9%		
		n	7,923	5,128			9,352	4,862		
		Mean (mmHg) (95% CI)	129.9 (129.6–130.2)	131.5 (131.0 – 132.0)	1.6	1.2%	126.3 (126.0 – 126.6)	125.6 (125.1 – 126.1)	-0.7	-0.6%
	**South-Asian**	% ascertained	100%	100%			100%	100%		
		n	93	110			67	77		
		Mean (mmHg) (95% CI)	125.7 (121.8–129.6)	124.8 (121.7–127.9)	-0.9	-0.7%	128.9 (123.7 – 134.1)	125.1 (121.4 – 128.8)	-3.8	-2.9%
**DBP**	**Caucasian**	% ascertained	100%	100%			100%	100%		
		n	7,924	5,130			9,357	4,865		
		Mean (mmHg) (95% CI)	80.4 (80.2 – 80.6)	81.8 (81.5 – 82.1)	1.4	1.7%	77.4 (77.2 – 77.6)	77.9 (77.6 – 78.2)	0.5	0.6%
	**South-Asian**	% ascertained	100%	100%			100%	100%		
		n	93	110			67	77		
		Mean (mmHg) (95% CI)	78.1 (75.8 – 80.4)	79.3 (77.4 – 81.2)	1.2	1.5%	78.3 (76.0 – 80.6)	77.5 (75.4 – 79.6)	-0.8	-1.0%
**BMI**	**Caucasian**	% ascertained	73.9%	88.4%			71.8%	86.9%		
		n	5,856	4,533			6,722	4,228		
		Mean (kg/m2) (95% CI)	25.84 (25.75 – 25.93)	26.65 (26.52 – 26.78)	0.81	3.1%	25.06 (24.95 – 25.17)	25.96 (25.77 – 26.15)	0.9	3.6%
	**South-Asian**	% ascertained	63.4%	87.3%			65.7%	84.4%		
		n	59	96			44	84.4		
		Mean (kg/m2) (95% CI)	24.83 (24.1 – 25.6)	25.94 (25.1 – 26.8)	1.11	4.5%	26.16 (24.9 – 27.40)	26.42 (25.23 – 27.61)	0.26	1.0%
**Smoking**	**Caucasian**	% ascertained	74.6%	88.5%			72.6%	87.0%		
		n	5,911	4,542			6,793	4,231		
		Mean (% current smokers) (95% CI)	51.9% (50.5 – 53.2)	43.6% (41.9 – 45.3)	-8.3	-16.0%	38.7% (37.5 – 39.9)	34.4% (32.7 – 36.1)	-4.3	-11.1%
	**South-Asian**	% ascertained	62.4%	86.4%			59.7%	80.5%		
		n	58	95			40	62		
		Mean (% current smokers) (95% CI)	25.9% (14.3 – 37.5)	27.4% (16.5 – 38.3)	1.5	5.8%	0% (0.0 – 0.0)	7% (0.0 – 14.0)	7	...

SBP: Systolic Blood Pressure, DBP: Diastolic Blood Pressure, BMI: Body Mass Index.

In regression analysis, adjusting for age and test year, South Asian men and women throughout the study period appear to have comparatively either lower or similar risk factor levels, with the exception of BMI for women (Additional File 4, Figures 1-10). In particular, South Asian men had significantly lower values of systolic BP [-4.91 mmHg (95% CI: -3.58 to -6.23)] and diastolic BP [-2.87 mmHg (-2.02 to -3.72)], with no significant difference in total cholesterol and BMI (Models 1a-b, Tables 3, 4). South Asian women had significantly lower systolic BP [-1.77 mmHg (-0.21 to -3.33)], diastolic BP [-1.87 mmHg (-0.92 to -2.82)], cholesterol [-0.24 mmol/l (-0.08 to -0.39 mmol/l)]; and higher BMI [+0.78 kg/m^2 ^(+0.25 to +1.30)]. South Asian men and women had significantly age-standardised lower prevalence of current smoking [29.0% (24.1%–33.9%)] and 1.8% (0.1%–3.5% respectively], compared to men and women of Caucasian origin [49.3% (48.7%–49.9%) and 36.6% (36.1%–37.2%) respectively].

**Table 3:** Mean annual change and overall difference in continuous risk factor levels by ethnic group stratum, adjusted for age and test year.

	**Men**				**Women**			
	
	**Mean**	**UCI**	**LCI**	**p**	**Mean**	**UCI**	**LCI**	**p**
**Systolic BP**								
***Model 1a (overall difference between South-Asian and Caucasian group)***								
Ethnic group difference^	-4.91	-6.23	-3.58	<0.001	-1.77	-3.33	-0.21	0.026
***Model 2a (trends by ethnic group)***								
Caucasian*	0.25	0.19	0.30	<0.001	0.02	-0.04	0.07	0.614
South-Asian*	-0.03	-0.46	0.39	0.874	-0.28	-0.79	0.23	0.277
***Model 3a (Time trend difference between South-Asian and Caucasian group)***								
Interaction ethnic group × time**	-0.27	-0.71	0.17	0.234	-0.29	-0.82	0.23	0.276
**Diastolic BP**								
***Model 1a (overall difference between South-Asian and Caucasian group)***								
Ethnic group difference^	-2.87	-3.72	-2.02	<0.001	-1.87	-2.82	-0.92	<0.001
***Model 2a (trends by ethnic group)***								
Caucasian*	0.19	0.15	0.22	<0.001	0.08	0.04	0.12	<0.001
South-Asian*	0.18	-0.11	0.46	0.228	-0.13	-0.44	0.17	0.387
***Model 3a (Time trend difference between South-Asian and Caucasian group)***								
Interaction ethnic group × time**	-0.22	-0.54	0.11	0.187	-0.22	-0.54	0.11	0.187
**Cholesterol**								
***Model 1a (overall difference between South-Asian and Caucasian group)***								
Ethnic group difference^	-0.04	-0.18	0.10	0.555	-0.24	-0.39	-0.08	0.002
***Model 2a (trends by ethnic group)***								
Caucasian*	-0.07	-0.08	-0.06	<0.001	-0.06	-0.07	-0.05	<0.000
South-Asian*	-0.08	-0.12	-0.03	0.001	-0.06	-0.10	-0.004	0.033
***Model 3a (Time trend difference between South-Asian and Caucasian group)***								
Interaction ethnic group × time**	-0.01	-0.06	0.04	0.763	0.02	-0.04	0.07	0.581
**BMI**								
***Model 1a (overall difference between South-Asian and Caucasian group)***								
Ethnic group difference^	-0.23	-0.59	0.14	0.223	0.78	0.25	1.30	0.004
***Model 2a (trends by ethnic group)***								
Caucasian*	0.10	0.08	0.11	<0.001	0.11	0.09	0.13	<0.001
South-Asian*	0.12	-0.02	0.25	0.083	0.00	-0.16	0.17	0.968
***Model 3a (Time trend difference between South-Asian and Caucasian group)***								
Interaction ethnic group × time**	0.05	-0.07	0.18	0.401	-0.12	-0.29	0.06	0.190

BP: Blood pressure, BMI: Body Mass Index, S-A: South-Asian

^adjusted for age, year, stratified by ethnic group

*adjusted for age, year and ethnic group

**adjusted for age, test year, ethnic group and interaction term "ethnic group × test year"

**Table 4:** Mean overall difference in probability of current smoking between ethnic groups.

	**Men**				**Women**			
	**OR**	**LCI**	**UCI**	**p**	**OR**	**LCI**	**UCI**	**p**

***Model 1b (overall difference between South-Asian and Caucasian group)***								
Ethnic group difference^	0.48	0.38	0.60	<0.001	0.03	0.01	0.08	<0.001
***Model 2b (trends by ethnic group)***								
Caucasian*	0.96	0.95	0.96	<0.001	0.97	0.96	0.98	<0.001
South-Asian*	1.01	0.93	1.09	0.858	1.42	0.99	2.04	0.059
***Model 3b (Time trend difference between South-Asian and Caucasian group)***								
Interaction ethnic group × time**	1.07	0.99	1.15	0.093	1.49	1.04	2.14	0.032

OR: Odds Ratio, UCI: Upper Confidence Interval, LCI: Lower Confidence Interval

^ adjusted for age and test year

* adjusted for age, stratified by ethnic group

**adjusted for age, test year, ethnic group and interaction term "ethnic group × test year"

### Comparisons of risk factor trends

Men of all ethnic groups manifested significant increasing trends in sysstolic BP [+0.23 mmHg/year (95% CI: +0.18 to +0.29)], diastolic BP [+0.18 mmHg/year (+0.15 to +0.22)], and BMI [+0.10 kg/m^2^/year (+0.08 to +0.11)]; and decreasing trends in total cholesterol [-0.07 mmol/l/year (-0.06 to -0.07)] (Models 2a-b, Tables 3, 4). Women of all groups manifested significant increasing time trends in diastolic BP [+0.08 mmHg/year (+0.04 to +0.11)] and BMI [+0.11 kg/m^2^/year (+0.09 to +0.13)], and decreasing time trends in total cholesterol (-0.06 mmol/l/year (-0.06 to -0.07)]. For smoking, overall there was a strong negative time trend in the probability of prevalence of current smoking for men [OR: 0.96 (0.95 to 0.96)]; and women [OR: 0.97 (0.96 to 0.97)]. Although current smoking prevalence was increasing in South-Asian individuals of both sexes, these trends were non-significant.

Empirically, compared with Caucasians, men and women of South-Asian origin did not have statistically significant differences in risk factor trends for systolic and diastolic BP, BMI and total cholesterol, as evidenced by overlapping confidence intervals for the *test year *co-efficient. Furthermore, for all continuous risk factors, the interaction term *ethnic group *X *test year *was non-significant, indicating that there is no statistically significant difference in time trend slopes between the groups (i.e. where significant differences between groups existed, they remain fixed during the study period) (Table 3, Model 3a). For smoking, there was a positive interaction between test year and ethnicity in women (p = 0.032), indicating a convergence in smoking prevalence rates between the two groups (Table 4, Model 3b), whilst for men, the p value was (0.093), which may be suggestive of an overall trend.

## Discussion

The study included UK individuals of middle age and with no previous diagnosis of hypertension, diabetes and cardiovascular disease. The findings indicate that during the 1990s and in the study setting, individuals of South-Asian origin did not have an adverse risk factor profile compared with the Caucasian ethnic group. In fact for some risk factors the inverse is true (i.e. more favourable risk factor levels among South-Asians). The findings also appear to show a similar pattern of time trends in risk factor levels for the examined five risk factors between Caucasian and South Asian groups. Individuals of South Asian origin are at higher risk of impaired glycose tolerance and diabetes, in itself a strong independent risk factor of cardiovascular disease, but no data on diabetic status were available for analysis. Nevertheless, the findings would contrast with the hypothesis that increased cardiovascular disease burden in UK South-Asians is due to higher level of exposure to conventional cardiovascular risk factors (other than impaired glucose tolerance and diabetes). Other hypotheses, such as greater genetic susceptibility to cardiovascular disease, may be relevant. Further research should also continue to explore the likely importance of novel and as yet unknown risk factors, and the likely relevance of lower risk of competing morbidity.

The lower levels of total cholesterol and smoking prevalence levels observed in the South-Asian group are in general agreement with previous findings from UK cross-sectional surveys [20,21], including the 1999 Health Survey for England (HSE) which focused on ethnic minority groups [4]. In particular, both the 1999 HSE and the present study indicate significantly lower prevalence of current smoking among South-Asian individuals particularly for women (although it should be noted that the 1999 HSE found higher smoking prevalence in one of the South-Asian ethnic subgroups – Bangladeshi men); similar or lower levels of cholesterol (particularly in relation to lower levels in South-Asian women); and higher levels of mean BMI in women. Conflictingly, a number of UK studies, previously expertly reviewed [22], have shown both higher (particularly in London) and lower levels of hypertension in South Asian compared to Caucasian populations -the present study being in broad agreement with the latter. In relation to BP, comparisons of the findings of the present study with the 1999 Health Survey for England area difficult, due to the variable findings in the latter survey, depending on South-Asian particular ethnic subgroup and sex.

The risk factor trends for total cholesterol, BMI and current smoking prevalence observed in this study are in agreement with those observed in UK population-based studies of the similar period (e.g. Health Survey for England (HSE) [3], Health Survey for Scotland [23]) i.e. reflecting downward trends for cholesterol and smoking status, and increasing trends for BMI. However, the lack of any considerable decrease in systolic and diastolic blood pressure for both men and women observed in the present study is in contrast to information from population-based epidemiological surveys [3,23]. This difference raises the question whether the difference can be due to bias, a "real" effect or chance. Selection bias is theoretically possible, as patients with known diagnosis of hypertension and other cardiovascular conditions were excluded from the study, unlike population-based surveys such as the HSE that randomly include both individuals with and without known diagnosis of hypertension (or cardiovascular disease). This hypothesis would have meant that the downward population trends in mean BP observed in HSE surveys of the same period are largely due to strong treatment effects and secular improvement in BP control among known hypertensive participants, and this is judged unlikely. Artefactual explanations are also likely, for example systematic differences in measurement progressively taking place during the study period (e.g. timing allowed to achieve a resting state, body posture, observer technique and training, number of BP readings, calculation of mean values from more than one readings).

A crucial question in relation to the generalisability of the study findings is whether screening participants were representative of the Stockport population of 35–60 year olds who were free of hypertension, diabetes and other cardiovascular disorders. However, if the rigour and accuracy with which individuals were identified and excluded because of known hypertension, diabetes and other cardiovascular conditions was differential between the two ethnic groups, then this potentially could have biased the findings, and this possibility cannot be dismissed with the data that are available.

The study used a computerised package to ascribe ethnicity. Reliance on the same software for identification of ethnic names has been proven not fully reliable before, particularly for South-Asian populations residing outside Northern England [15]. Nevertheless the computerised process of ethnic group assignment was supplemented by visual inspection of both the positive and the negative cases by two independent researchers of diverse ethnic background, which has been shown to improve specificity and specificity [15]. Overall, 1.23% of all cases were assigned South-Asian origin, which corresponds with estimates of the Stockport population of South Asian origin (all ages) of 1.12% in the 1991 and 2.1% in the 2001 Census [13]. In any case, any misclassification errors in the ascertainment of ethnicity would tend to make the two ethnic groups similar, and hence would have made true differences between ethnic groups more difficult to detect is such differences truly existed. Therefore although there is a possibility of undetected differences, those ethnic differences that have been identified in this study are unlikely to be biased by the method used to assign ethnicity.

The fact that Stockport has a relatively small South-Asian community, in combination with the fact that a relatively greater proportion of South-Asian population are younger than 30, is reflected in the small proportion of South Asians included in the study. It is theoretically possible that because Stockport is neither affluent nor deprived, Stockport South Asians are not representative of the wider South Asian communities in the UK.

An important limitation of the study is that the South-Asian ethnic group category contains an important degree of heterogeneity in terms of ethnic group, religion, culture and country of origin. Previous research has showed considerable heterogeneity in relation to exposure to cardiovascular risk factors between UK South-Asian ethnic groups [24]. Nevertheless, aggregation of all South-Asian groups has helped increase the statistical precision of estimates. In the 1991 census, within Stockport South-Asian individuals aged over 30 years of age, the relative proportion of those categorised as of Indian, Pakistani and Bangladeshi origin was 43.4 %, 49.5% and 7.1% respectively (data available from MIMAS, ). In the 2001 census a similar overall breakdown of South-Asian groups was observed (31.3%, 49.3% and 5.8%). It is therefore likely that the relative proportion of South-Asian subgroups in the dataset is a reflection of the proportions of subgroups of South-Asian origin in the community, but no further detailed study of such subgroups was possible. More importantly, it is unlikely that the observed time trends in risk reflect changes in the ethnic sub-group composition of the South-Asian group. Individuals belonging to ethnic subgroups are known to have an overall lower access rate to preventive interventions. We therefore acknowledge that it is possible that screening participants of South-Asian origin (and of some South-Asian subgroups in particular) may not be representative of the overall community. Unfortunately quantifying this potential problem is not possible with the available data.

## Conclusion

In a UK district the exposure of individuals of South-Asian origin to conventional cardiovascular risk factors excluding diabetes status was found to be overall either favourable or similar to individuals of Caucasian origin. Recent time trends in risk factor levels in South-Asians appear to mirror those observed in the Caucasian population, with the exception of smoking.

## Competing interests

The author(s) declare that they have no competing interests.

## Authors' contributions

GL had identified the research subject and defined the research question with support from and RFH. GL has developed the statistical methodology, with supervisory support by PMcE. PL and MH have over a number of years helped run the Stockport Cardiovascular Risk Factor Screening Programme and collect and collate data that enabled the analysis to be carried out. GL wrote the first draft of the manuscript. The work leading to this report has been carried out as part fulfilment for the study for the degree of MD, University of Manchester, for GL. RFH is his supervisor for the named degree and P McElduff his advisor.

## Acknowledgements

We would wish to acknowledge the help, advice and practical support of the following individuals: Professor Deborah Baker, Dr Stephen Watkins, Ms Jane Jefferson, Ms Jane Pilkington, Dr Gill Greenhough, Ms Jane Bowdenleigh, Ms Bernadette Ryan-Wooley, Ms Barbara Shallaker, Mr Dan Byrne. Dr Umesh Chauhan, Dr Robert (Mark) Hann UC and MH were responsible for the attribution of ethnic group.

## Pre-publication history

The pre-publication history for this paper can be accessed here:



## Supplementary Material

Additional File 1 'Supplementary information about the population coverage of the Stockport Cardiovascular Risk Factor Screening Programme 1989–1999, and its representativeness. This file includes information about population coverage (screening Programme uptake) among Stockport residents. Click here for file

Additional File 2 'Details about risk factor measurements'. Describes details about methods used in risk factor measurement. Click here for file

Additional File 3 'Risk factor ascertainment completeness (%) by ethnic group and sex, by test year period.' Describes the degree of ascertainment completeness by ethnic group. Click here for file

Additional File 4 'Age-standardised mean risk factor levels by ethnic group and sex, by test year period.' Self-explanatory. Click here for file
