# Supplementary material for: Comparative levels and time trends in blood pressure, total cholesterol, Body Mass Index and smoking among Caucasian and South-Asian participants of a UK primary-care based cardiovascular risk factor screening programme
Source: BMC Public Health. 2005 Nov 28;5:125. doi: 10.1186/1471-2458-5-125 (PMC1316876; doi:10.1186/1471-2458-5-125)
Supplement: Additional File 1 — 'Supplementary information about the population coverage of the Stockport Cardiovascular Risk Factor Screening Programme 1989–1999, and its representativeness. This file includes information about population coverage (screening Programme uptake) among Stockport residents. [file 1471-2458-5-125-S1.doc]

**ADDITIONAL FILE 1. Supplementary information about the population coverage of the Stockport Cardiovascular Risk Factor Screening Programme 1989-1999, and its’ representativeness.**

**1. Population coverage and representativeness**

To estimate population coverage of the screening programme by sex, as well as by ethnic group, one would have wished to have prospective information about the number of invitees who attended screening, by sex and ethnic group. However no such information was available about the number of persons “screened” as opposed to persons “invited”. Therefore, coverage by sex, and by deprivation group (as a surrogate marker of overall representativeness of screening participants, as denominator ethnic group data were not available) was assessed retrospectively and indirectly with the following method.

*Numerator information*

Programme participants (first screening) during the “prevalence round” 1989-1993 were used as the numerator. Deprivation group status was ascribed to each participant as described in Lyratzopoulos et al. 2005, BMC Public Health, <http://www.biomedcentral.com/1471-2458/5/32>, through quintiles of Census Enumeration District (ED) Townsend deprivation index score.

*Denominator information*

The number of Stockport residents aged 35-64 in each ED of the 1991 census was obtained from the “MIMAS Census Dissemination Unit CASWEB” search engine (University of Manchester). Data were stratified by sex 5-year age bands. The number of 60 year-old residents, was assumed to equate the number of residents in the age band 60-64 divided by 5. This was subsequently added to the number of individuals in all other 5-year old band (35-59) to produce the number of 35-60 year olds who were resident in each Stockport ED in the 1991 census –as this was the age group targeted by the Programme.

Using the same Townsend deprivation ED score quintile defining points as for the numerator, the 589 Stockport EDs were split into deprivation quintiles, and for each sex, the number of individuals aged 35-60 in each deprivation quintile was calculated.

*Coverage calculation (all individuals and by deprivation group)*

For each sex, the overall and deprivation group-specific coverage was subsequently calculated as the number of screening participants in each quintile divided by the respective number of individuals resident in the EDs of the respective quintile. The chi-squared test for trend was used to assess significance of any deprivation trends in screening coverage, using STATS DIRECT. The results are shown below.

**Additional File 1 Table 1: Population coverage (first screening episode) of the screening Programme 1989-1999**

|  | **Women**  **aged 35-60** |  | **Men**  **aged 35-60** |  |
| --- | --- | --- | --- | --- |
| **Deprivation Group** | **Coverage (%)** | **Test for trend** | **Coverage (%)** | **Test for trend** |
| **Affluent** | 76.1 | p=0.004 | 71.1 | p=0.190 |
| **2** | 77 |  | 71.1 |  |
| **3** | 79.5 |  | 73.7 |  |
| **4** | 78.6 |  | 72.5 |  |
| **Deprived** | 81 |  | 72.7 |  |
| **All** | 78.4 |  | 72.2 |  |

It is worth noting that true coverage would have been higher, as the denominator used in the above calculation includes all residents, i.e. even those that would have been excluded due to established / known cardiovascular disease or risk factors (see main text, Introduction).

**2. Socio-demographic changes in Stockport between 1991-2001.**

The main limitation of the above method relates to the fact that changes in the denominator (in terms of socio-demographic changes between 1989-1991 are ignored, in fact the method assumed that the socio-demographic composition remained “fixed” on 1991, and this year is representative of all other study years. This assumption is clearly inaccurate, but if the degree of inaccuracy is small, it may be still be an overall reasonable assumption.

To examine whether changes in the socio-demographic composition of Stockport (population age structure and ethnic groups) was considerable during the study period 1989-1999, we examined population differences between the two Census years 1991 and 2001, by sex and age, and by ethnic group. It was unfortunately not possible to obtain information on socio-economic changes, due to the different classifaction system used to assign socio-economic position in 1991 (Social Class) and 2001 (“National Statistics Socio-economic Classification) by the Office for National Statistics.

### Change in the age group 35-60, by sex

In relation to Stockport demography, looking at the 2001 and 1991 census figures, it is apparent that there has been very little change (reduction) in the 35-60 years age group, which was the group participating in the study (Additional File 1, Table 2).

**Additional File 1 Table 2: Number of Stockport residents aged 35-60 in 1999 and 2001, according to Census**

|  | **1991** | **2001** | **Absolute change**  **2001-1991** | **Proportional change (%)** |
| --- | --- | --- | --- | --- |
| **Men 35-60** | 52,559 | 50,862 | -1697 | -3.23 |
| **Women 35-60** | 53,638 | 51,806 | -1832 | -3.42 |
| **All persons all ages** | 284,395 | 284,422 | +27 | +0.01 |

(Source: MIMAS)

*Change in South-Asian ethnic group*

In relation to ethnic group, it can be seen that there has been a very considerable proportionally growth (near doubling) of the South-Asian origin group (“Sub-cont Asians/ Br. Asian)

**Additional File 1 Table 3: Stockport residents by ethnic group (all ages) in 1999 and 2001, according to Census**

| **All ages and sexes** | **1991** | **2001** |
| --- | --- | --- |
| Whites | 97.62 | 95.72 |
| **All Black** | 0.39 | 0.41 |
| **Sub-cont. Asians/ Br. Asian** | 1.12 | 2.10 |
| **Chinese** | 0.33 | 0.46 |
| **Other Asian** | 0.14 | - |
| **Other** | 0.38 | 0.28 |
| **Mixed groups** |  | 1.03 |
| **All ethnic groups** | 2.36 | 4.00 |
| **Total** | 99.98* | 100 |

*Number deviates from 100 due to rounding

(Source: MIMAS)

# Conclusion

There was an overall high level of population coverage, for both sexes. Coverage was highest among the most deprived women, but the overall difference between the most and least deprived group was small, both in absolute and proportional terms. This provides indirect evidence about the overall representativeness of study participants in relation to those in principle eligible for participation to the study. It was unfortunately not possible to apply this method to ethnic groups, as no age-specific information (35-60) was available at ED level.

During the period 1991-2001 (which overlaps considerably with the study period 1989-2001) there was overall little change in Stockport demography in terms of individuals in the 35-60 year olds age bracket.
