# Supplementary material for: Comparative levels and time trends in blood pressure, total cholesterol, Body Mass Index and smoking among Caucasian and South-Asian participants of a UK primary-care based cardiovascular risk factor screening programme
Source: BMC Public Health. 2005 Nov 28;5:125. doi: 10.1186/1471-2458-5-125 (PMC1316876; doi:10.1186/1471-2458-5-125)
Supplement: Additional File 2 — 'Details about risk factor measurements'. Describes details about methods used in risk factor measurement. [file 1471-2458-5-125-S2.doc]

**ADDITIONAL FILE 2**

**Details about risk factor measurements**

Total serum cholesterol was measured to 2 decimal points in mmol/L at the biochemistry laboratory of the Stepping Hill Hospital (a typical UK District General Hospital) by an enzymatic colorimetric assay using cholesterol esterase and cholesterol oxidase[[1]](#endnote-2). The Biochemistry laboratory participated in external quality assurance schemes. Fresh blood samples were sent to the laboratory and processed within 24 hours of sampling.

Physical measurements of systolic and diastolic blood pressure, weight and height, were measured with mercury sphygmomanometers, weight scales and stadiometers respectively, of variable types available at the Stockport General Practice surgeries participating in the scheme. Training for physical measurements was provided by a visiting nurse facilitator, employed by the Stockport Health Authority, whose role was to quality assure and co-ordinate the implementation of the screening activity. Height was measured to the nearest centimetre and weight to the nearest kilogram. Weight was measured with light clothing and without shoes. Body Mass Index was calculated by dividing weight in kilograms by height in meters squared. Systolic and diastolic blood pressure were measured according to a standardised protocol conforming to the 1987 “Recommendations on Blood Pressure Measurement” of the British Hypertension Society.[[2]](#endnote-3) Systolic blood pressure value was defined as the value at which the I^st^ Korotkoff sound becomes audible, and diastolic blood pressure as the value at which the V^th^ Korotkoff sound becomes inaudible, both recorded to the nearest 2mmHg (i.e. mercury column drop speed of 2mmHg/sec). A minimum of two measurements were made for each subject and inspection of the data suggests that values were rounded to the nearest 5 mm (data not shown). The mean of the measurements for each individual were used in the analyses.Error: Reference source not found

Smoking status was ascertained by direct questioning (self-reported). For the purposes of this analysis a dichotomous variable (current vs. non-current smoker) was created, by merging the “previous smoker” and “never smoker” categories.

1. Allain CC, Poon LS, Chan CS, Richmond W, Fu PC. Enzymatic determination of total serum cholesterol. Clin Chem. 1974;20(4):470-5. [↑](#endnote-ref-2)
2. British Hypertension Society. Recommendations on Blood Pressure Measurements. BMJ. 1987. [↑](#endnote-ref-3)
