# Supplementary material for: Comparative levels and time trends in blood pressure, total cholesterol, Body Mass Index and smoking among Caucasian and South-Asian participants of a UK primary-care based cardiovascular risk factor screening programme
Source: BMC Public Health. 2005 Nov 28;5:125. doi: 10.1186/1471-2458-5-125 (PMC1316876; doi:10.1186/1471-2458-5-125)
Supplement: Additional File 3 — 'Risk factor ascertainment completeness (%) by ethnic group and sex, by test year period.' Describes the degree of ascertainment completeness by ethnic group. [file 1471-2458-5-125-S3.doc]

ADDITIONAL FILE 3. Risk factor ascertainment completeness (%) by ethnic group and sex, by test year period.

Figure 1. Women participants, % ascertainment of Body Mass Index, total cholesterol and current smoking among Caucasian and South-Asian Individuals by year.

Figure 2. Men participants, % ascertainment of Body Mass Index, total cholesterol and current smoking among Caucasian and South-Asian Individuals by year**.**
