# Supplementary material for: Comparative levels and time trends in blood pressure, total cholesterol, Body Mass Index and smoking among Caucasian and South-Asian participants of a UK primary-care based cardiovascular risk factor screening programme
Source: BMC Public Health. 2005 Nov 28;5:125. doi: 10.1186/1471-2458-5-125 (PMC1316876; doi:10.1186/1471-2458-5-125)
Supplement: Additional File 4 — 'Age-standardised mean risk factor levels by ethnic group and sex, by test year period.' Self-explanatory. [file 1471-2458-5-125-S4.doc]

# ADDITIONAL FILE 4

**Age-standardised mean risk factor levels by ethnic group and sex, by test year period.**

Figure 1. Age-standardised mean total cholesterol levels in women by ethnic group, 1989-1999

Figure 2. Age-standardised mean total cholesterol levels in men by ethnic group, 1989-1999

Figure 3. Age-standardised mean SBP in women by ethnic group, 1989-1999.

Figure 4. Age-standardised mean SBP in men by ethnic group, 1989-1999.

Figure 5. Age-standardised mean DBP in women by ethnic group, 1989-1999.

Figure 6. Age-standardised mean DBP in men by ethnic group, 1989-1999.

Figure 7. Age-standardised mean BMI in women by ethnic group , 1989-1999.

Figure 8. Age-standardised mean BMI in men by ethnic group , 1989-1999.

Figure 9. Age-standardised mean prevalence of current smoking in women by ethnic group, 1989-1999.

Figure 10. Age-standardised mean prevalence of current smoking in men by ethnic group, 1989-1999.
